# Life on the margins: the experiences of sexual violence and exploitation among Eritrean asylum-seeking women in Israel

**DOI:** 10.1186/s12905-018-0624-y

**Published:** 2018-08-08

**Authors:** Tsega Gebreyesus, Zebib Sultan, Habtom M. Ghebrezghiabher, Wietse A. Tol, Peter J. Winch, Nadav Davidovitch, Pamela J. Surkan

**Affiliations:** 1 Johns Hopkins Bloomberg School of Public Health, 615 N. Wolfe Street, Baltimore, MD 21205 USA; 2 Eritrean Women’s Center, 2671 Wendee Drive, Apt. 1822, Cincinatti, OH 45238 USA; 3 Department of Health Policy and Management, School of Public Health, Faculty of Health Sciences, Ben-Gurion University of the Negev, POB 635, Beer-Sheva, 84105 Israel; 4 Johns Hopkins Bloomberg School of Public Health, 624 North Broadway, Hampton House (room 863), Baltimore, MD 21205-1996 USA; 5 Johns Hopkins Bloomberg School of Public Health, 615 North Wolfe Street Room E5533, Baltimore, MD 21205-2103 USA; 6 Johns Hopkins Bloomberg School of Public Health, 615 North Wolfe Street Room E5523, Baltimore, MD 21205-2103 USA; 7 Peter C. Alderman Program for Global Mental Health at HealthRight International, New York, NY USA

**Keywords:** Eritrea, Asylum, Policy, Forced migration, Marginalization, women’s health, Political and economic exclusion, Sexual violence and exploitation, Structural violence

## Abstract

**Background:**

Eritrean migrants in Israel, the majority of whom are seeking asylum, have limited access to institutional support. While the temporary group protection granted to Eritreans by Israel ensures that they are not deported, it does not confer permanent legal status, nor does it allow access to the formal work sector. This study qualitatively explores how political and economic marginalization increases the risk of sexual and other forms of violence as well as the exploitation of Eritrean women asylum seekers living in Israel.

**Methods:**

Twenty-five interviews with key informants, twelve individual interviews (six with men and six with women), and eight focus group discussions (four with men and four with women) were conducted among Eritreans of reproductive age in Tel Aviv, Israel. Qualitative data analysis was conducted using open, focused, and axial coding.

**Results:**

Participants reported that Israel’s restrictive immigration policies laid the foundation for the political and economic marginalization of asylum seekers. This manifested in limited access to institutional support during and after arrival, and hindered access to formal employment and its associated protections. The Israeli government’s decision to grant provisional status with a stipulation banning Eritreans from the formal work sector was perceived to create direct and indirect conditions for a heightened sense of structural vulnerability, particularly for women. Participants reported that this structural vulnerability increased the risk of sexual and domestic violence in addition to the risk for the exploitation of women asylum seekers.

**Conclusions:**

Israel’s immigration policies may contribute to women asylum seekers’ vulnerability to sexual violence upon arrival in their host country. These policies shape the social realities of women asylum seekers, potentially increasing their risk of violence and exploitation during their time in Israel. This study provides an example of the effects of political and economic marginalization on violence against women, a concept that may apply to other settings globally.

**Electronic supplementary material:**

The online version of this article (10.1186/s12905-018-0624-y) contains supplementary material, which is available to authorized users.

## Background

Globalization, political instability and increasing economic and social disparities between low, middle and high-income countries have led to growing waves of transnational migration [1]. Throughout the migration experience, women asylum seekers endure multiple hardships including sexual violence, the absence of social support, and the inability to avail themselves of the protection of their countries of origin or any other institutional or legal recourse [2–4]. Increased transnational migration also has challenged nation-states and societies to reassess their definitions of citizenship and their willingness to integrate refugees politically, economically and socially [1, 5]. This tension is reflected in the degree of access to state-provided services, like health care to migrants - including those with claims for asylum [1, 5].

These issues have become particularly salient for Eritreans in Israel where there has been substantial anti-African asylum-seeker sentiment reflected in the media and in the law [6, 7]. Since 1951, less than one-percent of asylum seekers in Israel have been granted official political asylum (refugee recognition) [8]. While over ninety-percent of Eritrean asylum seekers are granted refugee status in host countries around the world [9], until 2015, Israel only recognized two asylum seekers as refugees [10, 11]. This is because the Israeli government offered Eritrean asylum seekers ‘group protection’ while encouraging self-deportation though detention and financial support for leaving the country [12]. In 2013, the government allowed processing of individual claims for refugee status [12]. However, for the vast majority, the government has either rejected or failed to act upon their request. This has left Eritreans in a legal limbo in which they are neither granted official refugee status and the accompanying rights to the social welfare system and the formal work sector, nor are they being deported [6, 13].

According to the United Nations [14], approximately 4000 Eritreans flee their country each month [15–17]. Some leave for the refugee camps in northeastern Ethiopia and eastern Sudan, while others try to reach North Africa, the Middle East, Europe, Canada and the United States [15]. The majority of the hundreds of thousands of Eritreans leaving have fled to countries in the region including Ethiopia, Sudan, Israel, Egypt, and Kenya [15, 16]. While the initial exodus of Eritreans was predominantly male, in recent years large numbers of Eritrean women began leaving the country, often following their husbands and other family members [13]. Informal estimates indicate that 7000 of the approximately 35,000 Eritreans asylum seekers are women, the overwhelming majority of whom arrived in Israel after 2009 [18].^1^

Israel has been a preferred destination, and until 2012, Eritrean refugees often sought to move there from Libya, Sudan, Ethiopia and Egypt [19]. This was because, until recently, the forced repatriations at the border and “voluntary” repatriations of Eritreans from Israel were far lower than those reported from Libya, Sudan, and Egypt [16]. In addition, Israel was perceived to offer a more hospitable social and economic environment than neighboring countries [16, 20]. While the vast majority of Eritreans who have entered the country since 2007 do not have permission to work officially, the money earned working informally is enough to sustain themselves, help pay their debts, pay the diaspora tax (2%) [21] if they fear for their families’ safety or assets, and send remittances to their families in Eritrea [13, 20].

The challenges faced by asylum seekers in Israel as a result of their temporary status have been well documented by humanitarian organizations, activists, scholars and the affected community itself [6, 7, 11, 12, 22]. Temporary protected status, and its stipulations banning asylum seekers from working in the formal sector [13], may result in political and economic marginalization that affects the lives of asylum seekers and shapes the risks they incur, particularly for women. Most research on sexual violence and exploitation of migrant women, including studies conducted in Israel, focus on sex trafficking in the host country, experiences during war in the home country, and experiences of violence in refugee camps [23–38]. Little research has been conducted on the sexual violence and exploitation experienced by asylum-seeking women in Israel, despite the evidence that this is a particularly vulnerable group [33, 39]. This study seeks to extend previous research by addressing how ‘temporary protected status’ and its impact on work opportunities and living conditions influences vulnerability to sexual violence and exploitation.

The organizing framework for this paper is Zimmerman’s model for migration and health [40]. The model conceptualizes contemporary migration as a “multistage cycle that can be entered into multiple times, in various ways, and may occur within or across national borders”. According to this theory, migration includes the following stages: 1) pre-departure; 2) travel; 3) destination; 4) interception; and 5) return. Zimmerman’s theory states that understanding of the events at each stage is critical to comprehending the cumulative impact of migration on health and for strategizing appropriate interventions [40].

We focus on the destination stage of the framework for two reasons. First, there is a dearth of research on asylum seekers and their vulnerability to violence and exploitation in their host countries in the industrialized world. Second, sexual violence and corresponding negative health outcomes that occurred during the travel stage may be compounded by additional negative experiences at the destination stage.

In order to provide a more comprehensive understanding of the “built in” barriers that shape the experiences of the study population, we applied the theoretical lens of structural violence [41, 42]. This lens provides a framework for understanding the “structural vulnerability” [43] of marginalized populations and that individual agency is constrained by the wider risk environment [44]. Finally, exploring these women’s vulnerabilities to abuse, and their potential connection to national immigration and asylum policy, may lay the groundwork for future research and evidence-based policy changes at local and state levels [33].

## Methods

We conducted both in-depth individual interviews (“IDIs” with key informants and Eritrean community members) and focus group discussions (“FGDs” with Eritrean community members) (see Questionnaires for IDIs and FGDs Additional file 1). The IDIs enabled us to understand the breadth of individual experiences; while the FGDs served to gather information about social norms. Conducting both IDIs and FGDs allowed us to compare and contrast perspectives [45–47] of key informants and members of the Eritrean asylum-seeking community in Israel [45–47].

With the input of collaborators at Johns Hopkins and Ben Gurion University investigators, we developed guides for IDIs and FGDs. Then, with the help of a team of experts from the NGO and Eritrean community in Israel, we pre-tested and refined the guides among Eritrean community members and NGO workers. All study participants were at least 18 years old. In Phase II all participants were of reproductive age (between 18 and 49 years old). We obtained informed written consent from key informants and written or oral consent from asylum seekers (those who feared providing a signature provided oral consent) prior to each interviews. We took detailed notes and audio-recordings of all Phase I and Phase II IDIs and FGDs. Finally, we developed a referral list of existing services in the event that participants requested support (social, psychological or otherwise).

### Phase I

The study was conducted in two phases. Phase I (December, 2012 to April, 2013) focused on barriers to contraceptive care-seeking from a health systems perspective. During Phase I, IDIs with 25 key informants (20 Israelis, one American and four Eritreans) were conducted by the lead author. The lead author is fluent in Tigrinya and English and conducted four interviews with Eritrean key informants independently. The lead author, with the help of an Israeli research assistant fluent in Hebrew and English, conducted the remaining 21 interviews with Israeli participants in English (and Hebrew when necessary) (see Table 1).

**Table 1:** Participants by Phase

Study design	Methods	Study sample	Sample size
Phase I	Semi-structured IDIs	Key informants (governmental and non-governmental health care and other service providers)	*N* = 25 individual interviews
Phase II	Semi-structured IDIs	Eritrean men and women	*N* = 12 Individual interviews (*n* = 6 male; *n* = 6 female)
	Semi-structured Focus Group Discussions		*N* = 8 focus groups (4 with males and 4 with females; 4–8 people per group) *N* = 44 participants

Phase I participants worked with the Eritrean asylum-seeking community in Israel (one of the eligibility criteria). Phase I participants were recruited from a list provided by various domestic and international NGOs, the Ministry of Health, private clinics and from researchers at an Israeli university [48]. Interviews lasted approximately one to two hours.

Our Phase I interviews investigated three core topics: reasons for unwanted pregnancies; accessibility of contraception services for Eritrean women in Israel; perspectives on vulnerability of Eritrean women to sexual violence and exploitation en-route to and in Israel; and the general experiences of Eritrean asylum seekers navigating the Israeli health system. Recruitment ended after reaching data saturation (i.e. no new themes emerged during data analysis) [49].

### Phase II

Phase II (April 2013 to September 2013) focused on Eritrean community members’ perspectives on both family planning and their access to contraceptive services in Israel. Phase II consisted of 12 IDIs (six with women and six with men) and eight FGDs (four with male and four with female members of the Eritrean asylum-seeking community, total *n* = 44). All Phase II IDIs were conducted in a private room in Tigrinya. Phase II participants were eligible if they were Eritreans of reproductive age (between 18 and 49 years old), who arrived in Israel after 2007 and lived there at the time. In-depth interview participants were not eligible to participate in FGDs (see Table 1).

In-depth interview participants were recruited from the PHR-Israel “Open Clinic” an Israeli NGO health facility that provides health services to the uninsured. The research team identified the “Open Clinic” as a recruitment site through the ethnographic mapping of health facilities where asylum seekers frequently obtain healthcare services (one of two humanitarian clinics serving this population in Israel). All interviews lasted between one and three hours. The interview guide investigated three core topics: knowledge of contraception methods, barriers to contraceptive careseeking, and vulnerability to unwanted pregnancies.

FGD participants were both purposively sampled (through contacts with community activists, researchers and staff at the Open Clinic) and snowball sampled [48]. Due to concerns about detainment and deportation as well as internal political tensions within the Eritrean asylum-seeking community, the initial set of purposively sampled FGD participants were asked to recruit up to seven other people they felt comfortable sharing with to participate in a group session, for a total of four to eight members per group [50, 51]. FGDs (held separately for women and men to observe any social norms that differed by gender) lasted between one to three hours. Refreshments were provided for participants.

The lead author conducted FGDs in locations that participants considered discrete and safe (e.g. back rooms in participants’ stores, homes and NGO meeting rooms). The FGDs explored social norms regarding fertility, unwanted pregnancy, and family planning.

#### Translation and data analysis

Professional transcription companies (one based in the United Kingdom and the other in Ethiopia) not affiliated with the study team fully transcribed all interviews. Interviews conducted in English were transcribed directly; interviews conducted in Tigrinya were translated and then transcribed into English by professionals fluent in both languages and then a select number were back-translated by the lead author. The lead author conducted all qualitative analyses from the onset of data collection until June 2014.

The lead author employed open, focused and axial coding using ATLAS.ti software for all data collected during Phases I and II (Atlas.ti, Berlin, Germany) and discussed themes with the group. Axial coding of focused codes was used to identify the themes described. Throughout the coding process written memos informed the conceptual development of codes and themes [52]. Member-checking with a sub-sample of key informants and Eritrean participants provided an opportunity to share findings and to assess the trustworthiness of results [46].

Ethical approval for this study was obtained from both the Ben Gurion University of the Negev, the Physicians for Human Rights Israel, and the Johns Hopkins University Bloomberg School of Public Health Institutional Review Boards.

This paper explores experiences of sexual violence and exploitation of Eritrean women in Israel. Analyses on barriers to contraceptive careseeking and violence en-route to Israel are reported elsewhere [53, 54].

## Results

Participants identified the political exclusion resulting from provisional status and the consequent economic exclusion from the formal workforce as the two principal factors that shaped their risk of sexual violence and exploitation as asylum seekers in Israel. The men quoted in this paper did not personally experience the sexual violence and exploitation described. Thus the information that they provide must be interpreted with the knowledge that they are third-party voices. The women who are quoted alternated between first- and second person narration. The second-person narration may be used as a tool for distancing themselves from the trauma of the violence that they were threatened with, or they personally incurred.

### Consequences of political marginalization

#### Provisional status

The majority of individual and focus group participants discussed stressors resulting from their status as provisional de facto refugees in Israel. Some associated their provisional legal status with increased risks of sexual violence. Before 2012, Israeli policy towards Eritrean asylum seekers was to accord them temporary protection which meant that they were not kept in detention, but neither were they provided any social support [6, 12, 13]. All participants reported that, under Israel’s informal policy of settlement in Tel Aviv they were given pre-purchased tickets and sent directly from the detention facility to Tel Aviv where they were left to fend for themselves. Some participants said that direct method of settlement exposed women who had no existing community of support and protection to possibilities of sexual violence and exploitation. One participant recounted her experience:

*We took the bus and then they drop you off here [Tel Aviv]. Where do you go? To Levinsky park [located near the central bus station in Tel Aviv], where else. (Eritrean woman IDII7).*

Another female participant explained the situation generally:

*If a woman came here and she was alone, she would have begged to stay with anyone. If she did this, it shows those she is asking that she has no family. There was always a chance that they [fellow asylum-seeking men that take her in] would rape her. (Eritrean woman IDI5).*

Another individual participant described her personal experience arriving in Tel Aviv as a woman without support below:

*I arrived here by bus with five other women. One of them had a husband in Tel Aviv who came to pick her up. The remaining four of us decided to stick together. Some men from our community saw us at the bus stop and offered to buy us dinner. We decided to go as a group. They offered us shelter at their home for the night. We had nowhere else to go, so we went with them. When we reached the house there were many men in a very tiny room with four beds... A group of men decided to take one woman for themselves and I said to them ‘I am going to shout, I am a virgin, I don’t know [have ever had sex with] any man. After all the things I have gone through coming here, you want to rape me? I am going to scream.’ I screamed and screamed. One of the men raped one of my friends in front of us… I screamed ‘what are you doing?’ and he hit me over the head with a phone until I bled. As she was being raped, she screamed to me to shut up so that they wouldn’t kill us but I couldn’t. I shouted and threatened to go to the UN to report them. We didn’t sleep that night...a neighbor called the police and the men fled. When the police came, I was bleeding. (Eritrean woman IDI2).*

After 2012, new arrivals were sent to detention rather than being directly settled and that change of policy had implications for women and their children who had arrived beforehand. One female participant explained how her husband’s detention impacted her vulnerability:

*I can say I am better off [than my husband in detention] because I can work and come home and also raise my child but I am alone in this country with no one to support me and sometimes I would prefer for us to switch places…I am so alone. Here, even if you are married there are men that check you out… there are men that would check you out while you are carrying your baby on your back … I am fearful that worse things could happen. There are people that get close to you [a woman], and they pretend to be good to you… but in the end they end up hurting you. You may know how to avoid your enemy, but what do you do if the man who hurts you is a friend? With the struggles here [in Tel Aviv] I prefer for him [my husband] to be here instead of me because he is a man and no one would touch him. As a girl there is violence, murder, rape and so much more. (Eritrean woman IDI10).*

#### Consequences of economic marginalization

##### Informal work sector

Israeli labor policy bans asylum seekers from working in the formal sector, but employment in the informal economy is tolerated [6, 12, 13]. All participants confirmed that Eritrean asylum-seeking women work in the informal work sector, often as domestic workers, to sustain themselves and their families. The majority of participants reported that sexual harassment and, in some cases, sexual violence was common among women working as housekeepers, cleaners or babysitters. One male participant explained his observations of the sexual harassment of women in the workplace:

*I hate it. They [Israeli employers] ask them [Eritrean women] to have sexual intercourse with them... They [employers] threaten to fire them from work. When they threaten the women it makes them think ‘how am I going to survive without work’... we don’t have the right to work in Israel... she is not thinking about herself... she is thinking about keeping her job so she feels she may have to do this [sleep with employers]. It happens. (Eritrean man IDI6).*

Another participant explained:

*There are many stories like this …there are many rapes. They take them, rape them and pay them money. But since she wants to live and make money, she would still go back there to clean and work. (Men’s focus group 2, participant 2).*

All participants reported that one way of finding work was by standing in certain areas around Levinsky Park and on certain roads in the mornings. One male participant explained his perception of the particular risk for women of seeking informal work (*chik chak*) in this way.

*So when these women [Eritrean women] get here [to Israel], they go and stand in the park to wait for employers looking for cheap labor. If it’s an Eritrean woman, for example, he [a potential Israeli employer] would tell her, ‘Come with me I can help you find work.’ They pick them up and the women go because they don’t have other options. Then the Israeli would take them to work and lock them up and sexually abuse them. (Eritrean man IDI9).*

A female focus group participant confirmed:

*It’s work and the women need jobs. If a girl waits on the street looking for a job [informal work], they [Israeli employers] take them to a secluded area -- to a place where you can’t even scream for help and do as they please [sexually abuse the woman]. Sometimes they tell them the job is in a house and they take them to do it there. (Women’s Focus Group Discussion 3, participant 1).*

Another focus group participant explained:

*Two years ago I saw a story posted that two men raped a woman. They worked chik chak [informal daily work] and they took her in to a building and she stayed there… they kidnapped her. She stayed there for two weeks and she didn’t know the language or directions. They took her phone and she stayed there for two weeks. (Women’s focus group discussion 2, participant 3).*

Many participants reported that the sexual violence and exploitation can arise even if the employment relationship originally was for domestic services. The lack of opportunity for other employment and the absence of institutional support for legal work creates ongoing conditions of vulnerability. A male participant who witnessed the sexual exploitation of women shared his understanding of the situation:

*At the end it all comes down to financial security. There are two women that I know. They used to work with me. The man who employed us was Russian-Israeli. He took one girl to clean for him in another area and she went with him. He did what he did to her [raped her] and when he finished he tried to get a third woman to come home with him to clean. The other two women said no. I was there at the time so I supported the remaining women in their decision. We all got fired after that. (Men’s focus group 2, participant 2).*

Another focus group participant described women’s perceptions that reporting abuse to authorities made no difference when working in the informal sector:

*I think even if you get raped and you tell the police nobody takes you seriously … chik chak [informal work] is the worst … (Women’s focus group 4, participant 3).*

### Prostitution

Many participants reported that exclusion from the formal work sector drives some Eritrean women to engage in prostitution which is another way that provisional status exposes women to increased risk of sexual violence. Many participants reported that Eritrean women provided sex to Eritrean and Israeli men in exchange for resources. One focus group participant explained:

*When men offer them [Eritrean women who become prostitutes] that kind of money, they get attracted to it. They don’t think about the consequences. They focus on the money that is being offered to them. They think to themselves, ‘why work all day when I can get money this way?’ …then they agree to sleep with the men... Then an unplanned pregnancy happens and another man comes to have sex with them. They end up selling their souls for money. (Women’s focus group discussion 2, participant 2).*

A female individual participant connected Eritrean women involved in prostitution with economic and political marginalization:

*Yes they sell their bodies. What choice do they have? What other work do they have? They give sex to anyone that gives them money. If you escaped your country and you don’t have a visa what other choice do you have? They usually begin bartending with a bit of prostitution on the side but because of the bar fights, some women shifted into full time prostitution. Honestly, like I said there are women that can’t work...and so they work by selling their bodies and acquire a disease and sometimes an unwanted pregnancy happens. They go to doctors and pay lots of money to get rid of these pregnancies and, in order to pay for the abortion, they prostitute themselves on top of the pregnancy. Do you understand? They have to spend the night with someone so that they can get the abortion the next day. (Eritrean woman IDI2).*

### Crowded living conditions

Many participants cited crowded living spaces as yet another factor connected to economic and political marginalization which increases women’s vulnerability to sexual violence and exploitation. While the social norm of living with others, the gender imbalance in the community, and the lack of known and trusted sources of social support were indicated by participants to result in many women sharing living quarters, often with men who they do not know, the majority of participants agreed that the underlying reason for shared housing was economic pressure. Many participants stated that while the sharing of spaces made life in Israel affordable, the crowded and often mixed-gender environment, in which there are usually many more men than women, often increased women’s vulnerability to sexual violence. A male key informant explained his understanding of the risk:

*Okay, you’re living with eight or ten men. A relative brings a woman to live with your group. So she’s living among eight men who’ve been here for five years with no women at all. Sometimes the women are forced [to have sex] by one of the men. Sometimes they are not forced but the situation creates pressure. Bringing a woman into a living environment where there are eight men is a way of forcing her right? Even married couples have to live in this situation. One problem that married couples face is that one of the men living in the home may ask or pressure the wife to sleep with them while her husband is at work. Do you understand me? She may be able to protect herself and her marriage for 99 out of 100 days but there is always that chance that something can happen. Something can happen one day. (Eritrean Man KI25).*

One female focus group participant described single women’s vulnerability to sexual violence:

*To live as a single woman is very stressful. They [men] won’t leave you alone unless you are married … the single women can’t afford to live alone, it’s expensive to cover food and all... So she lives with a group, either with men or women. It’s often very difficult to find other single women to live with so they live with men who are their relatives or who are from the same region. These women get asked for sexual favors all the time, and the men force themselves on them … it’s very difficult … the women are ashamed of the fact that they were raped so they won’t tell. They are ashamed to say that a man from their hometown raped them. (Women’s focus group 1, participant 5).*

### Domestic violence

Many participants also reported an increased risk of domestic violence, including wife-murders, which they attributed in part to the effect of political and economic marginalization of Eritrean men. Many participants noted that women are caught between relying on men for the family’s livelihood and the need to protect themselves from violence:

*My husband, he would hit me with an electric wire and I would bleed. Once the police came and saw the blood. They [the police] said as long as he [husband] is alive he is not getting out of prison. Honestly, even though he had hit me and I lost my child [a pregnancy] I didn’t want him to be in prison because I have another child. I would rather my husband help me financially but the government won’t let him out. Women don’t call the police because they don’t expect to get murdered. You expect the fight to pass. You try and tolerate it but then it gets out of control. You understand that the men are stressed too. You pray that with time the violence will pass but it is beyond you. The circumstances of our lives are bad. The men have stress here [in Israel]. It has been almost six years since Eritreans came to this country and the government hasn’t taken care of us … they don’t have visas for us. We don’t have rights like Eritrean refugees in other countries … there is oppression here. After washing dishes all day some men come home and accept it [their fate]… and some don’t. Those men that don’t say to themselves ‘why aren’t our rights respected like others [citizens]?’ They want to go to libraries and study in schools like Eritreans who are accepted as citizens in other countries. Here it is not like that. The men are doing badly here. (Eritrean woman IDI2).*

Another male focus group member connected political and economic pressures to wife-murders in the Eritrean community:

*I think the stress is the main problem. No Eritrean has hope in this country. For example, they [the Israeli government] don’t tell you anything that is coming for you, for example whether you will be able to get papers [more permanent status] after a certain amount of time. There is no sign of improvement and so there is a lot of stress. It could be my perception but honestly most Eritreans work 12–18 h days. If a man and a woman fight there is no time or energy to solve the problem. Are you going to rest and call for people [other community members who can mediate] to settle it? As a result these things [disputes] get more serious with time. So the main problem is the difficult situation for Eritreans in this country. I have never heard of killing your wife in Eritrea. It’s my first time hearing that a man killed a woman. It’s brought up by the stress, the psychologists say that too, it’s the stress. (Men’s focus group 3, participant 1).*

## Discussion

The core finding of this paper is that there may be a connection between state-level policy and women asylum seekers’ vulnerability to sexual violence upon arrival in their host country - the “destination” stage of Zimmerman’s model [40]. Our results suggest that women’s provisional status and relegation to the informal work sector heightens their vulnerability to sexual violence (e.g. from employers and fellow-countryman) and further hinders their ability to take legal action. These factors also perpetuate the economic challenges these women face, exacerbating their impoverishment, driving involvement to prostitution and crowded living conditions and consequently putting them at further risk.

Zimmerman’s model provides a framework for understanding that the experiences of sexual violence and exploitation are cumulative over multiple stages of movement [40, 55–59]. Zimmerman’s model does not however delve into the political and economic environments in the destination country and their role in increasing risk of sexual violence [40]. Using the theory of structural violence^2^ [41, 42] we were able to explore how restrictive immigration policy in Israel may have had repercussions in terms of the exigency that women have to seek necessary employment in unregulated sectors where they may be more vulnerable to sexual violence and exploitation, with little recourse to institutional support or protection. The unpacking of Zimmerman’s “destination stage” through the lens of structural violence [42] enabled us to explore the ways in which politically-driven social inequalities contributed towards increasing women’s risk of violence and exploitation.

Political and economic marginalization in Israel affected all Eritrean participants in the study (including men). The majority of the 81 participants (37 individual interviews and 44 focus group participants) whose views are reported here claimed that Israeli policy granting them only provisional status (and the resulting barriers to employment in the formal sector) contributed to their marginalization within Israeli society. Israel’s policies leading to structural vulnerability (e.g. not being able to have work permits) were perceived to render Eritrean women (in particular) vulnerable because they lack trusted, stable and secure social support, affordable shelter, or the ability to earn a legal income enabling them to live independently. The informal policy of directly sending asylum seekers to Tel Aviv without providing additional support (in effect prior to 2012), was described as an example of a practice that resulted in many women arriving in Tel Aviv without money, connections or shelter. The lack of refugee assistance was reported to leave new arrivals dependent upon strangers for food, lodging, and employment. Participants stated that one of the repercussions of built-in exclusion from the social welfare system was an increase in sexual violence perpetrated against Eritrean women asylum seekers.

Policies giving rise to political and economic exclusion also were perceived to have repercussions for women’s structural vulnerability, referring to the vulnerability that results from economic exploitation and gender and racial discrimination within society [43]. The decision of the Israeli government to grant provisional status with a stipulation banning Eritreans from the formal work sector may, according to participants, create conditions that foster sexual abuse. Most Eritrean asylum-seeking women in our study worked as domestic workers in the informal work sector and did not have official refugee status, which limited their recourse for legal protection.

Our findings support previous research suggesting that migrants are at high risk of sexual victimization at every stage of their migration experiences including the time after their arrival at the country of destination [3, 4, 43, 60–64]. For asylum seekers who are not afforded protection under international refugee law in their host countries, risk of abuse is considerably higher than for those who are granted official refugee status [33, 39, 65, 66]. This was relevant to the experiences of all of the Eritrean asylum seekers in our study as none of them were afforded the full protections of refugee status. All participants in the study had conditional release visas (temporary protection) that banned them from working in the formal work sector.

Studies in the Middle East, Europe, Asia and Africa, have found that migrant women without the legal status necessary for formal protection in the work sector are often sexually and psychologically abused by their employers [33, 65, 67–69]. Our results reinforce these findings as women working in the informal sector indicated an increase in their risk of sexual violence by their employers. Similar to our finding that some Eritrean women asylum seekers in Israel resort to prostitution, research conducted in Europe found that marginalized African migrant women often rely on prostitution as a source of income [34]. While we do not know of other research connecting crowded living conditions, migration and risk of sexual violence, results of this study suggested that this combination may influence risk of sexual violence and exploitation of asylum-seeking women. It may be argued that these risks are a form of structural violence that is embedded in the policies governing asylum seekers’ legal rights to settle as refugees.

We also explored the impact of political and economic marginalization on intimate partner violence. Research conducted in the United States and Europe found that the high prevalence of intimate partner violence in migrant communities is partly rooted in political and economic pressures affecting them [70–72]. In addition to placing stress on a marriage, women who do not have legal status in a country or have other forms of non-permanent status do not receive necessary institutional support when experiencing domestic violence as they may fear for their own or their partner’s detention or deportation [71, 73]. Our findings illustrate a similar situation for Eritrean women with provisional status, in which political and economic marginalization was suggested to serve as key stressors that fueled spousal abuse.

A major strength of this study is that it is the first of its kind to investigate Eritrean asylum-seeking women’s vulnerability to sexual violence and exploitation in Israel. Qualitative data about the experiences of Eritrean women in Israel allows for an in-depth exploration of the various circumstances of vulnerability in which Eritrean asylum-seeking women find themselves and their understandings of the factors creating their vulnerability. The use of information gleaned from interviews and focus group discussions with Eritreans allowed for a comprehensive understanding of the effects of political and economic exclusion and its implications for the sexual vulnerability of Eritrean women living there.

The inclusion of male Eritrean participants’ perspectives added an additional perspective, uncommon in most studies of violence against women. Male participants provided supportive detailed information about the experiences of sexual violence and exploitation faced by Eritrean women living in Israel. The willingness of men to discuss these traumatic events may be related to them not having been personally abused (men may have less fear of being re-traumatization by sharing their narratives). While the information is indirect, it complemented the information shared by the women themselves. The use of male testimonies also highlights the psychological stressors that men experience when they observe this abuse. The women themselves shifted between first- and second-person narration. This might have been a mechanism for them to distance themselves from information that could be traumatic and stigmatizing.

The study also has limitations. The focus on the testimonies of eighty-one participants (based on 25 key informant interviews as well as 12 interviews and 8 focus groups with community members) may not reflect the variety of experiences of Eritrean asylum-seeking women living in Israel and it is possible that some women may not experience sexual violence or exploitation. The overwhelming majority of employed Eritrean women in Israel and in our study work as cleaners in the informal sector, therefore experiences may be different for those in other sectors. For those who have experienced it, this study may not comprehensively describe all of the possible circumstances of risk connected to their marginalization.

An important solution to the challenges facing this population would be to provide a more sustainable legal status to asylum seekers and to authorize them to work formally without interruption. This would likely lead to economic stability and to the reduction of violence as well as access to health insurance. Another potential solution to the risk of sexual violence and exploitation these women endure would be to provide asylum seekers with social support in the form of government-sponsored safe lodging. Information about the importance of reporting sexual abuse to the police irrespective of the perpetrators background should be shared with the asylum-seeking community.

## Conclusions

Our results suggest that the nature of the status granted to Eritreans in Israel has implications for the ability of Eritrean women to stabilize their livelihoods and establish themselves in their employment and households free from abuse or sexual pressure from other residents in group living situations. This structural violence has implications not only for their risk of sexual violence and exploitation, but also, depending on the nationality and level of authority of the perpetrator, their recourse for legal or other institutional protection. These circumstances appear to create an environment of impunity for the abuse of vulnerable populations within a context in which non-marginalized groups are highly protected. Areas of future research might include an exploration of factors influencing the wellbeing of male Eritrean asylum seekers (also highly marginalized), but may face distinct challenges. The implications of these findings are not unique to Israel and may shed light on sources of challenges facing similarly marginalized women in other parts of the world in similar circumstances. In line with the underlying theory of Zimmerman’s model, future research could more explicitly explore whether experiences at the destination stage of the migration cycle were compounded by experiences of sexual violence during the pre-departure and travel stages.

## Additional file

Additional file 1: Questionnaires: The additional file is comprised of the questionnaires used during the study for the purposes of data collection. These questionnaires were used during all key informant interviews, individual interviews and focus group discussions. (DOCX 121 kb)

## Abbreviations

FGD: Focus group discussion
IDI: In-depth Interview
NGO: Non-governmental Organization
UNHCR: United Nations High Commissioner for Refugees

## Footnotes

1 The population size is an estimate and may have decreased due to self-deportation in recent years.

2 Structural violence is defined here as enduring “ubiquitous social structures, normalized by stable institutions and regular experience” [74] that result in disparate access to resources, political power, education, health care, and legal protections [75]. This social organization, ingrained in the political and economic structures, results in the continuance of inequality [76]
