# Supplementary material for: Life on the margins: the experiences of sexual violence and exploitation among Eritrean asylum-seeking women in Israel
Source: BMC Womens Health. 2018 Aug 8;18:135. doi: 10.1186/s12905-018-0624-y (PMC6083583; doi:10.1186/s12905-018-0624-y)
Supplement: Supplementary file 1 — Questionnaires: The additional file is comprised of the questionnaires used during the study for the purposes of data collection. These questionnaires were used during all key informant interviews, individual interviews and focus group discussions. (DOCX 121 kb) [file 12905_2018_624_MOESM1_ESM.docx]

### Questionnaire

### Key informant interviews: Unintended Pregnancies among Eritrean asylum seekers

**Interviewer name:** _______________________________________________________________________________________________

**Interview date:** ________________________________ **Interview location:** ___________________________________________

***Complete consent form (explaining purpose of study, confidentiality of information***

***and voluntary nature of interview) prior to beginning the interview.***

**Module 1: Demographic Information**

| **MODULE 1: DEMOGRAPHIC INFORMATION** | | | |
| --- | --- | --- | --- |
| **Number** | **Question** | **Instructions** | **Coding** |
| 1. | Nationality?  ____________________ | Record actual nationality. | 1. Eritrean  2. Israeli  99. Other: ___________ |
| 2. | Age? ______________ | Record actual age. | 1. >20 2. 21-25  3. 26-30 4. 31-40  5. 40-50 6. 50+ |
| 3. | Gender? (circle one)  Male / Female |  | 1. Male  2. Female |
| 4. | Occupation  _________________________ | Record exact occupation on the left and circle the category in which it falls on the right. | 1. Health practitioner (physician, nurse, PA etc.)  2. Social Worker  3. NGO representative  4. Government worker  5. Community member  6. Community leader  7. UNHCR representative  99. Other: __________ |
| 5. | Mother tongue?  __________________ | *What language did the participant speak while growing up at home?* | 1. Afar  2. Amharic  3. Arabic  4. Bilen  5. Hedareb  6. Saho  7. Kunama  8. Nara  9. Tigre  10. Tigrinya  11. Hebrew  12. English  13. Fur  99. Other: ___________ |
| 6. | Other languages spoken?  ________________________________  ________________________________ | *List as many as the participant answers.* | 1. Afar  2. Amharic  3. Arabic  4. Bilen  5. Hedareb  6. Saho  7. Kunama  8. Nara  9. Tigre  10. Tigrinya  11. Hebrew  12. English  13. Fur  99. Other: ___________ |
|  |  |  |  |
| 8. | How many years of experience do you have working with the Eritrean asylum-seeking populations? |  | 1. >1 year  2. 1-5 years  3. 5+ years  99. Other: _____________ |

| 9. | What is the highest level of education that you have completed?  _______________________________ | *Record highest level obtained.* |  |
| --- | --- | --- | --- |

| **Number** | **Question** | **Instructions** | **Coding** |
| --- | --- | --- | --- |
| 10. | What was your occupation in your home country?  ______________________________ | Only ask if the interviewee is a member of the refugee community or an expatriate. Record occupation. |  |
| 11. | What is the average age of women becoming pregnant in this population?  ______________________________ |  | 1. > 18 years  2. 18-25 years  3. 25-30 years  4. 30-35 years  5. 35-40 years  6. 40+ years |
| 12. | Does your organization deal with family planning? |  | 1. Yes 2. No |

#### Qualitative open-ended questions

**MODULE 2: REPRODUCTIVE HEALTH CARE SEEKING**

1. What is the legal status of the women with whom you work?
2. Could you describe some challenges that these women face in their daily lives?
   1. Probe on how legal status influences their access to health care.
3. Could you describe the factors that empower women in these communities?
   1. Probe on resources that enable women to deal with the challenges of being in Israel.
4. Could you describe any challenges that you have observed in the access to family planning for these women in the health system?
   1. Probe on structural level challenges, discrimination, miscommunication
5. Could you describe any challenges that you have observed in the access of family planning for these women at the community and individual level?
   1. Probe on gender relations, community dynamics, religion, cultural practices
6. Could you describe the demand for family planning in your experience?
7. To what extent do you understand the family planning desires of this population?
8. Could you describe any challenges in accessing family planning unique to this population as compared to Israeli’s or other migrants
9. Could you describe the reproductive health services available to women who were trafficked through the Sinai when they arrive to Israel?
10. To what extent do you know about informal provision of family planning services?
    1. Probe: Physicians involved in the provision of such care using informal/poor quality practices and also members of community providing reproductive health care
11. Could you describe how family planning provision is monitored for quality assurance?
12. To what extent do you consider unwanted pregnancies to be a concern for this population?
13. Could you describe the factors that put women at risk for these pregnancies?
14. Please describe the consequences for bringing unwanted pregnancies to term for these women and their partners and families.
    1. Probe: Social, legal and economic consequences separately.
15. Could you describe any challenges in communication between providers and patients?
16. Please describe the consequences of miscommunication that you have observed in the provision of reproductive health services.
    1. Probe on how miscommunication influences the demand for family planning
17. Could you describe a potential solution to the challenge of reproductive health care provision for these migrants?
18. Could you describe the reasons for the absence of a governmental solution to health care for the Eritrean community?

a. Probe on the evolution of the current health provision for asylum-seekers

1. Is there anything else you would like to add that you think may be helpful for me to know in learning about access to reproductive health care and/or any of the topics discussed above?

### Questionnaire: Individual interviews with Eritrean asylum seekers

**Interviewer name:** _______________________________________________________________________________________________

Translator present: yes or no

Translator name: _______________________________

**Interview date:** ________________________________ **Interview location:** ___________________________________________

***Complete consent form (explaining purpose of study, confidentiality of information***

***and voluntary nature of interview) prior to beginning the interview.***

**Module 1: Demographic Information**

| **MODULE 1: DEMOGRAPHIC INFORMATION** | | | |
| --- | --- | --- | --- |
| **Number** | **Question** | **Instructions** | **Coding** |
| 1. | Nationality?  ____________________ | Record actual nationality. | 1. Eritrean  2. Sudanese  3. Ethiopian  99. Other: ___________ |
| 2. | Age? ______________ | Record actual age. | 1. >20 2. 21-25  3. 26-30 4. 31-40  5. 40-50 6. 50+ |
| 3. | Gender? (circle one)  Male / Female |  | 1. Male  2. Female |
| 4. | Occupation  _________________________ | Record occupation. | 1. Health practitioner (physician, nurse, PA etc.)  2. Social Worker  3. NGO representative  4.Hospitality  5. Construction  6. Janitorial services  7. Childcare  8. Day laborer  6. Business owner  7. Student  99. Other: __________ |
| 5. | Do you have a work contract? |  | 1. Yes 2. No |
| 6. | Do you have health insurance through your work? |  | 1. Yes 2. No |
| 7. | If yes, what type? |  | 1. Israeli national health insurance 2. Private self- sponsored insurance 3. 99. Other: _________ |
| 8. | Do you have any other form of health insurance in Israel? |  | 1. Yes 2. No |
| 9. | If yes, what type? |  | 1. Israeli national health insurance 2. Private self- sponsored insurance   99. Other: _________ |
| 10. | Mother tongue?  __________________ | *What language did the participant speak while growing up at home?* | 1. Afar  2. Amharic  3. Arabic  4. Bilen  5. Hedareb  6. Saho  7. Kunama  8. Nara  9. Tigre  10. Tigrinya  11. English  12. Fur  99. Other: ___________ |
| 11. | Other languages spoken?  ________________________________  ________________________________ | *List as many as the participant answers.* | 1. Afar  2. Amharic  3. Arabic  4. Bilen  5. Hedareb  6. Saho  7. Kunama  8. Nara  9. Tigre  10. Tigrinya  11. Hebrew  12. English  99. Other: ___________ |
| 12. | What city/town/rural area were you born in? | Record location. | 99. Other: ___________ |
| 13. | What is your marital status? |  | 1. Married 2. Single 3. In a relationship 4. Divorced 5. Widowed 6. Other: _________ |
| 14. | If married or in a relationship, is your partner in Israel? |  | 1. Yes 2. No |
| 15. | Do you have children? |  | 1. Yes 2. No |
| 16. | If yes, how many children do you have? |  | 99. Other: ________ |
| 17. | Are your children in Israel? |  | 1. Yes 2. No |
| 18. | What is your living situation in Israel?  ____________________________ | If other please record living situation. | 1. Rent a room with friends/family 2. Rent a room alone 3. Rent a room with spouse/partner 4. Stay with friends/family 5. Stay with stranger 6. Stay with partner 7. Stay in shelter   99. Other __________ |
| 19. | Do you have your own income? |  | 1. Yes 2. No |
| 20. | How much do you make per month? |  | 1. Less than 2,000 2. Between 2000-4000 3. Between 4000-6,000 4. Greater than 6,000 |
| 21. | Do you send money home? |  | 1. Yes 2. No |
| 22. | How often do you send money home? | Record how often. |  |
| 23. | If yes, what proportion of your income is sent home? |  | 1. less than 10%  2. 10%-20%  3. 20-30%  4. Greater than 30% |
| 24. | How many years have you lived in Israel? |  | 1. >1 year  2. 1-3 years  3. 3-6  4. 6+ years_____________ |

| 25. | What is the highest level of education that you have completed?  _______________________________ |  | 1. No formal education 2. Pre-primary, 3. Primary, 4. Middle, 5. Secondary 6. Tertiary   99. Other: __________ |
| --- | --- | --- | --- |

| **Number** | **Question** | **Instructions** | **Coding** |
| --- | --- | --- | --- |
| 26. | What was your occupation in your home country?  ______________________________ | Record occupation. |  |
| 27. | Did you travel alone to Israel? |  | 1. Yes 2. No |
| 28. | What countries did you travel to en route to Israel? |  | Country 1: ____________  Country 2: ____________  Country 3: ____________  Country 4: ____________  Country 5: ____________  Country 6: ____________  Country 7: ____________  Country 8: ____________ |
| 29. | How much time did you spend in each place? |  | Country 1: ____________  Time (months): ________  Country 2: ____________  Time (months): ________  Country 3: ____________  Time (months): ________  Country 4: ____________  Time (months): ________  Country 5: ____________  Time (months): ________  Country 6: ____________  Time (months): ________  Country 7: ____________  Time (months): ________  Country 8: ____________  Time (months): ________ |
| 30. | What is the average age of becoming sexually active in your population? | Record in years. |  |
| 31. | What is the average age of women becoming pregnant in your population?  ______________________________ |  | 1. > 18 years  2. 18-25 years  3. 25-30 years  4. 30-35 years  5. 35-40 years  6. 40+ years |
| 32. | Please list all of the forms of contraception with which you are familiar. | Check all mentioned. If they mention something not listed write in other category. | 1. Oral contraception 2. Injectables 3. Implants 4. IUDs 5. Female Condoms 6. Male Condoms 7. Foam 8. Withdrawal 9. Abstinence   99. Other: __________ |
| 33. | Have you ever used any form of contraception? |  | 1.Yes  2. No |
| 34. | If so, what types? |  | 1. Oral contraception 2. Injectables 3. Implants 4. IUDs 5. Female Condoms 6. Male Condoms 7. Foam 8. Withdrawal 9. Abstinence   99. Other: __________ |
| 35. | Are you currently using any form of contraception? |  | 1.Yes  2. No |
| 36. | If so, what types? |  | 1. Oral contraception 2. Injectables 3. Implants 4. IUDs 5. Female Condoms 6. Male Condoms 7. Foam 8. Withdrawal 9. Abstinence   99. Other: __________ |
| 37. | If you have a partner in Israel, are they currently using any form of contraception? |  | 1.Yes  2. No |
| 38. | If so, what types? |  | 1. Oral contraception 2. Injectables 3. Implants 4. IUDs 5. Female Condoms 6. Male Condoms 7. Foam 8. Withdrawal 9. Abstinence   99. Other: __________ |
| 39. | Were you educated on its use? |  | 1.Yes  2. No |
| 40. | If so, by whom? |  | 1.Health care provider  2. Friends  3. Family  3. religious leaders  4.Other community members  5. Spouse  99.Other:________________ |
| 41. | What is your preferred method? |  | 1. Oral contraception 2. Injectables 3. Implants 4. IUDs 5. Female Condoms 6. Male Condoms 7. Foam 8. Withdrawal 9. Abstinence   99. Other: __________ |
| 42. | Have you or anyone you know experienced an unplanned pregnancy? |  | 1.Yes  2. No  99. Other: ________ |
| 43. | Have you or anyone that you know experienced an unwanted pregnancy? |  | 1.Yes  2. No  99. Other: _______ |

#### Qualitative open-ended questions (IDI)

**MODULE 2: REPRODUCTIVE HEALTH CARE SEEKING**

**Access to family planning**

1. Could you describe for me the availability of family planning here in Israel?
   1. Probe: where it is available, who it is meant for, what types are available, informal provision, stores that sell condoms
2. Could you describe the preferred method of contraception on average in the community?
   1. Probe: women and men separately,
3. Could you describe the reasons that this/these method/s are preferred?
4. Could you describe the quality of the family planning services that are available to you?
   1. Probe: patient-provider communication, translator, what is lacking, what could be improved
5. Could you describe the support that you have when you need contraception here in Israel?
   1. Probe: who do you go to discuss your options, who directs women to the health facilities, translation
6. From your experience, could you describe access to contraception in your home country.
   1. Probe: Are there any barriers to access? What makes it difficult? Economics? Knowledge? Availability?
7. Could you describe the process of accessing family planning and other forms of reproductive health care in Israel?
   1. Probe: Are there any barriers to access? What makes it difficult (Economics? Knowledge? Availability? Spousal support? Religion? Social norms?)? Are there different norms for married and unmarried women? Has that changed in Israel?
8. Could you describe the main difference between access to contraception and other forms of family planning here and in your home country?
9. Could you describe ways in which access to family planning services could be improved for women and men in your community?

**Unwanted Pregnancies**

1. Could you describe how your role as a man/woman in your community has changed in Israel?
   1. If yes, probe to assess how the new context has influenced relationships.
2. Could you describe the decision-making power that women have in their relationships?
   1. Probe: For example, would a woman have the power to purchase goods, where to live, whether or not to have a baby
3. Could you describe what would make a woman vulnerable to unwanted pregnancies?
   1. Probe: decision-making within a marriage, living environment, demographic imbalance, Israeli employers, other migrants
4. Could you describe the reasons that a pregnancy would be unwanted?
5. Could you describe the consequences of an unwanted pregnancy?
   1. Probe: economic, social, legal consequences for mothers and fathers separately
6. Could you describe the pros and cons of contraceptive use in your community
   1. Probe: from the perspective of women, men and children separately
7. Could you describe what women/couples/families do in light of an unwanted pregnancy?
8. Is family planning accessible through informal networks?
   1. Yes or No
   2. Probe: If so, please describe where they are found, who provides them and peoples experiences with them.
9. Could you describe any experiences that you or anyone that you know has had with the reproductive health care system in Israel?
   1. Probe: cultural and linguistic barriers to communication

10. Is there anything else you would like to add that you think may be helpful for me to know in learning about access to reproductive health care and/or any of the topics discussed above?

### Unintended Pregnancies among Eritrean Asylum-seekers

### Interviews with Asylum-seeker

**Moderator name: _______________________________**

**Translator present: Yes or No**

**Translator name:_____________________________________**

**Focus Group Discussion Location:_______________________**

***Complete consent form (explaining purpose of study, confidentiality of information and voluntary nature of interview) prior to beginning the interview.***

**FGD Guide**

*Research Question:* What are the barriers and facilitating factors to the access and utilization of contraception among Eritrean Asylum Seekers in Israel?

*Estimated Time: 1.5* hour

**Module 1: Ask of each FGD participant separately prior to the start of the group discussion**

| 1. | What is the average age of becoming sexually active in your population? | Record in years. |  |
| --- | --- | --- | --- |
| 2. | What is the average age of women becoming pregnant in your population?  ______________________________ |  | 1. > 18 years  2. 18-25 years  3. 25-30 years  4. 30-35 years  5. 35-40 years  6. 40+ years |
| 3. | Please list all of the forms of contraception with which you are familiar. | Check all mentioned. If they mention something not listed write in other category. | 1. Oral contraception 2. Injectables 3. Implants 4. IUDs 5. Female Condoms 6. Male Condoms 7. Foam 8. Withdrawal 9. Abstinence   99. Other: __________ |
| 4. | Have you ever used any form of contraception? |  | 1.Yes  2. No |
| 5. | If so, what types? |  | 1. Oral contraception 2. Injectables 3. Implants 4. IUDs 5. Female Condoms 6. Male Condoms 7. Foam 8. Withdrawal 9. Abstinence   99. Other: __________ |
| 6. | Are you currently using any form of contraception? |  | 1.Yes  2. No |
| 7. | If so, what types? |  | 1. Oral contraception 2. Injectables 3. Implants 4. IUDs 5. Female Condoms 6. Male Condoms 7. Foam 8. Withdrawal 9. Abstinence   99. Other: __________ |
| 8. | If you have a partner in Israel, are they currently using any form of contraception? |  | 1.Yes  2. No |
| 9. | If so, what types? |  | 1. Oral contraception 2. Injectables 3. Implants 4. IUDs 5. Female Condoms 6. Male Condoms 7. Foam 8. Withdrawal 9. Abstinence   99. Other: __________ |
| 10. | Were you educated on its use? |  | 1.Yes  2. No |
| 11. | If so, by whom? |  | 1.Health care providers  2. Female friends  3. Family  3. religious leaders  4.Other community members  5. Spouse  99. Other:___________________ |
| 12. | Have you or anyone you known experienced an unplanned pregnancy? |  | 1.Yes  2. No  99. Other: ________ |
| 13. | Have you or anyone that you know experienced an unwanted pregnancy? |  | 1.Yes  2. No  99. Other: _______ |

#### Qualitative open-ended questions (FGDs)

**Module 2: Focus group discussion**

*Probes to continue conversation*: Can you tell me more about that? Does anyone disagree? Does anyone agree? Can we see a show of hands? I see you are nodding (or shaking your head) in agreement (or disagreement), what part of that statement did you agree (or not agree) with? Is there anything further on this topic that someone would like to add? What is our consensus opinion on this topic before we close it out?

**BARRIERS AND FACILITATORS**

1. **Could you describe any reasons that women would be vulnerable to an unwanted pregnancy?**

**Probes:**

- Ability to access contraceptive information or services (linguistic competency in Hebrew or English, health worker attitudes)
- Demographic composition of living spaces
- (gender imbalance)
- Type of relationship (power structure of these relationships)
- Work environment
- Economic pressures (dependence on men to help survive, financial insecurity)
- Rape
- Social expectations to marry and give birth
- Fear of detention
- Limited social support

1. **Could you describe some of the consequences of an unwanted pregnancy?**

**Probes:**

- Impact on social standing within social networks (perception of unwanted pregnancies)
- Employment
- Economic standing
- Strain on relationship (if in a relationship)
- Stigma if not in a relationship
- Psychological stress due to insecure nature of their stay in Israel
- Pressure to remain in an undesired relationship

1. **Could you describe how life in Israel is different from life at home?**

**Probes:**

- Employment (control of finances)
- Change in the male to female ratio
- Social structure (lack of familial support, absence of gatekeepers)
- Linguistic barriers
- Power dynamics within relationships

1. **Could anyone describe any barriers to contraceptive access in your home country?**

- Financial constraints
- Health infrastructure (accessibility of facilities, availability of supplies, geographic distance)
- Gender (gender expectations of pregnancy as a mark of womanhood, respect conferred)
- Social stigmatization of sexual intercourse for unmarried women

1. **Could anyone describe barriers to contraceptive access in Israel?**

**Probes:**

- Discrimination due to ethnicity
- Discrimination due to race
- Discrimination due to religion
- Discrimination due to migrant status
- Financial constraints (Costs of procurement)
- Health infrastructure (accessibility of facilities, geographic distance)
- Gender (gender expectations of pregnancy as a mark of womanhood, respect conferred)
- Linguistic barriers (miscommunication)
- Employment (stability of employment in light of pregnancy or health concern**)**
- Parity (does the number of children that a woman has affect her desire to access contraception?)
- Age
- Self-efficacy
  - Do I feel capable of accessing contraception?
- Contraception's benefits outweigh its risks?
- Fear of side effects (physical (infertility) and emotional
- Fear of being "found out"
- Contraception is effective?
  - Contraception a good use of funds?
- Ability to use consistently
- History of behavior: previously used contraception
  - What was last experience of contraception like and how does that affect attitudes today?
- Knowledge of contraceptive methods
- Current health status

1. **Could you describe anything that would make access to contraceptive use easier?**

**Probes:**

- Communal support, knowledge, streamlined list of service providers,

1. **Could you describe if there are any methods that are preferable to others?**

**Probes:**

- Ask why each method mentioned is preferable and why others are not
  - Taste, smell, secrecy,
- Traditional methods vs. modern methods
